# How does short video use generate political identity? Intermediate mechanisms with evidence from China’s small-town youth

**DOI:** 10.3389/fpsyg.2023.1107273

**Published:** 2023-01-27

**Authors:** Jing Qin, Quanqingqing Du, Yuanbing Deng, Bowen Zhang, Xiaohui Sun

**Affiliations:** ^1^School of Journalism and Communication, Zhengzhou University, Zhengzhou, China; ^2^Mu Qing Research Center of Zhengzhou University, Zhengzhou, China; ^3^Institute of Communication Studies, Communication University of China, Beijing, China; ^4^College of Management, Shenzhen University, Shenzhen, China

**Keywords:** youth behavior, social media use, political identity, cultural identity, political trust

## Abstract

**Introduction:**

China’s Small-town Youth is an important social class connecting urban and rural development. Its ideological status is closely related to social stability and development so that the study of political identity of small town youth in China has become an important topic.

**Methods:**

Based on survey and in-depth interviews, this paper investigates the intermediate mechanism of short videos use on political identity of China’s Small-town Youth.

**Results and discussion:**

The study found that the use of short videos by China’s Small-town Youth has a significant positive impact on political identity, and media trust and political trust play a complete mediating role between the use of short videos and political identity. Cultural identity as the main content of China’s political ideology propaganda, cannot affect political trust. This research provides theoretical basis and practical enlightenment for the systematic understanding of the spiritual world and political beliefs of China’s Small-town Youth, and for exploring how to enhance their political identity through short videos use.

## 1. Introduction

In recent years, discussions on topics such as “Houlang” and “small-town swot” (Swot here refers to a person who spends too much time studying) in China have become popular on the Internet. The former refers to the emerging generation of young people with great potential for development, while the latter refers to the young students who were born in small towns, studied hard from childhood, and were good at taking exams but lacked certain vision and resources. It’s axiomatic that the new generation of young people has become a hot topic of concern. Among them, the young people in small towns, which connect urban and rural areas, constitute a significant link in creating an “olive-shaped social structure” in today’s Chinese society. Young people in small towns are those who were born in counties and towns in or below the third and fourth tier areas, grow up in their hometown, and then move to big cities, provincial capitals, and surrounding cities (Qin, 2021). According to China’s National Bureau of Statistics, the number of China’s small-town youth has reached 227 million (Zhang and Chen, 2022). Driven by the Chinese government’s rural revitalization strategy and development policy of urban-rural integration, the scale of China’s small-town youth is still expanding and becoming a rising and prominent group in Chinese society. However, with the rapid economic development in first-tier cities and the acceleration of the new urbanization process, small-town youth face a large gap between their living situation and consumption potential, resulting in their vulnerability to the negative hostage of the technological revolution and economic capital at the spiritual level, which hinders the formation of positive ideological and political concepts. Previous studies have shown that the class identity, social justice, and social trust of China’s small-town youth are generally low (Zhao and Sun, 2019).

The class inequality of small-town youth is projected in the media discourse, resulting in their social and cultural leadership being at a disadvantage as well. The group is demonized and stigmatized, and they develop more negative political attitudes. The emergence of short videos provides an opportunity to improve this dilemma. Due to the low threshold of content production and strong adaptability of scenes, short videos reduce the deficit of discourse (Peng, 2019). A large number of China’s Small-town Youth gather on short video platforms to construct self-image and express emotions independently (Liu and An, 2019), and the empowerment of self-expression contributes to the promotion of their sense of identity. Although there are still various kinds of communication chaos and value risks in the short video platform, with the change of new audio-visual media technology, the impact of short video on the political identity of China’s Small-town Youth has also generated more possibilities. In the meanwhile, political identity, as a psychological manifestation of political standpoint, is believed to be closely related to public trust (Hernandez and Minor, 2020). Trust in the short video platform and the political system may play a role in the interaction between the use of short videos and the construction of political identity.

Based on the above discussion, this study examines the impact of short video use on political identity of China’s Small-town Youth, and the intermediate role that media trust and political trust may play in this process. Moreover, this study aims to systematically understand the spiritual world and political concepts of young people in small towns in China, better guide their political values and explore an effective way to enhance their political identity through short videos.

## 2. Literature review and hypotheses

### 2.1. The political efficacy of the media

As politics is expanding and broadening from “emancipatory politics” to “life politics,” the role of the media in this transformative process turned necessary (Verstraeten, 2004). With it regarding the importance of media as source of information about politics and society, the autonomy of media institutions from other social and political institutions are usually guided by political identity in media logic or political logic (Strömbäck and Esser, 2014, p. 375–403). Media is able to play a definite role in post-truth politics, as so-called post-truth era is now involved in a by-product of populism on the mass media (Salgado, 2018).

The media also plays a role in political perspectives between different social groups. According to the research, the richness of resources owned by different social classes is related to the depth and breadth of media use, and there is also a significant gap between their media access and use ability (Murdock and Golding, 2004, p. 244–260). For example, people with lower socioeconomic status have a lower level of media use; In terms of media presentation, most of the media’s attention is focused on elite members from the “high level” of society, creating and further deepening class stereotypes (Kendall, 2008). These factors will affect the life feeling and social mentality of individuals in the group, and thus affect their political identity.

Political identity is a key factor influencing political identity (Miner et al., 2021). Scholars consider the relationship between media use and political identity, and generally believe that as a media with the function of spreading ideology, it has fully realized its political function, and all kinds of media have a certain effect on shaping identity (Jennifer and Cynthia, 2020). This way of thinking includes two negative effects and positive effects: first, media use crowds out people’s input in social public life and weakens political identity; Secondly, the media can improve the political cognitive ability and promote the public’s political trust and sense of identity. Comparing the two differences, it can be speculated that “trust” may be the key to transforming the negative effects of media use and enabling it to promote political identity. Effective government depends on the consent of the governed and the ability of citizens to get along with each other under a certain degree of mutual understanding (Newton et al., 2018, p. 37). Therefore, the effective political function of the media may also be achieved through the two paths of citizens’ acceptance of the media and their psychological support for the political system, media trust and political trust should be taken into account.

### 2.2. The relationship between short video use and political identity

Political identity refers to the individual’s affiliation to political units, geographical regions and groups in social life, as well as the subjective cognition of certain political theories, views and propositions (Rosenbaum, 1984). It also shows the process of internalizing these into psychological character and emotional will, and externalizing them into political literacy and political behavior (Gentry, 2018). It includes three dimensions, as political interest identity, political system identity and political value identity (Kong, 2007). It is the basis of political identity for individuals to obtain benefits from the political community, so that they can obey and recognize the system of political parties and governments. Then their emotions will be satisfied and transformed into belief and affirmation of mainstream ideology and values.

Media technology is a critical factor affecting people’s political cognition and attitude. In recent years, the Internet has provided more selective space for the public to contact and interact with political information. Hence, the impact of media use in the Internet era on public political consciousness is complicated. Some studies have shown that ICTs have ensured the construction and maintenance of people’s daily political identity (Garrett et al., 2012). Social media has created a mass base, technical support and practical mode for the construction of political identity, but at the same time, there may exist risks such as blocking the construction of mainstream ideology due to multiple identities (Lu et al., 2016).

Since the public opinion field on short video gradually emerged in 2016, Chinese government at all levels and mainstream media have settled in the short video platform. Now, short video platform has been put an important position for the dissemination of mainstream values. Previous studies have shown that government affairs of multiple central level units have formed an effective transmission of positive emotions, which is conducive to people’s sense of identity with the country and government (Zhang and Yin, 2019). Short videos drive users’ re-transmission behavior through emotional communication, which is conducive to improving the public’s political identity (Zhao and Li, 2022). Furthermore, short videos record and perpetuate rural culture, reflect on public issues such as the plight of the countryside, and greatly influence the ideology and value identity of small town youth. Therefore, this study puts forward the following research hypothesis:

H1: The use of short video has a positive effect on the political identity of China’s Small-town Youth.

### 2.3. The intermediate effect of media trust

As an important factor affecting people’s political ideas and attitudes, trust has always attracted academic attention. Media trust refers to the reliability of media perceived by the public (Burgoon and Hale, 2010). The research shows that, media trust originates from the attributes of the media itself and the public’s contact with media, and it increases along with the media use frequency (Johnson and Kaye, 2009). Wang (2017) found that there is a strong positive correlation between media trust and political psychology. Lu and Duan (2015) further pointed out that media trust, as an intermediate variable, affects the influence of the Internet on young people’s political attitudes. It is apparently that the communication effect of the content of short video platform is affected by the trust of public media. The higher the trust, the easier it is to accept and recognize the information and values transmitted by short video. Therefore, the following hypothesis is proposed:

H2: The media trust of China’s Small-town Youth plays an intermediate role in the influence of short video use on political identity.

### 2.4. The intermediate effect of political trust

Political trust refers to citizens’ belief or confidence in the government or political system operation to produce results consistent with their expectations (Miller, 1974), which directly affects people’s political attitudes. Different types of media use have different effects on people’s political trust. The use of political news by traditional media has positive and significant effects on political trust, while the use of online political news has significant negative effects (Chadwick, 2013). At present, various government departments in China release government information through the platform of short video, trying to enhance public political trust and optimize the effect of political communication (Zhou and Lu, 2019). It has been confirmed that political trust has a significant positive effect on college students’ political party identification (Saarinen et al., 2019), promoting the process of political identity in real political life (Patrizia, 2004). The government or relevant institutions can enhance political identity by building political trust in cyberspace (Zmerli, 2014). Based on this, this study puts forward the following research hypothesis:

H3: The political trust of China’s Small-town Youth plays an intermediate role in the influence of short video use on political identity.

## 3. Research methods

### 3.1. Sample

According to the definition of All-China Youth Federation, youth mainly refers to adult citizens under the age of 40 years old, so the age selection criteria is 18–40 years old. In terms of the sampling method, this study adopts both offline and online ways with non-probability judgmental sampling method and snowball method to collect data from China’s Small-town Youth aged 18–40 who were born in counties and towns at the third and fourth tier or below. Finally, 525 questionnaires are effectively collected.

**(1)** On sample size, Schiffman and Kanuk (1991) and Yan et al. (2018) clarify the basic rules of sample size determination for the sampling method: (1) A sample size larger than 30 but smaller than 500 is suitable for most studies; (2) when there are more variables, a sample size of 10 times or more than 10 times the number of variables is optimal. According to this rule, there are 34 questions measuring core concepts in this study, and a sample size of 400 or more would be appropriate. In this paper, over 400 valid questionnaires were returned, which meets the sample size requirement. **(2)** On sampling technique, this study adopts both offline and online methods with the non-probability judgmental sampling method and the snowball method to collect data from China’s small-town youth. According to the administrative division of the Chinese government, there are four levels of urban hierarchy: provincial cities, provincial capitals, prefecture-level cities, and county-level cities. In this paper, “small-town youth” is defined as being from county-level cities and below. Considering the vast size of China and the varying levels of economic development in many county-level cities and below, this paper uses a non-probability judgmental sampling method to conduct the questionnaire survey, enhancing the representativeness of the sample. So we select the samples from a number of county-level cities and below in eastern, central, and western China, which is a strong representation of the group.

Through the descriptive statistical analysis of the samples by the data statistical analysis software SPSS25.0, the sample distribution is as follows: the education level of the survey participants is concentrated in junior college and undergraduate (82.5%); Communist Youth League members accounted for the majority (75.4%); male accounted for 49.1%, female accounted for 50.9%; 76.1 percent of them have lived or studied in big cities, and 29.1 percent of them are young people from small towns who have returned home from cities. The sample distribution is basically consistent with the public report on China’s Small-town Youth (Southern Weekend and PPDAI, 2018), which is in line with the characteristics of China’s Small-town Youth, so the sample can be considered reasonable.

In order to test the reliability and validity of the questionnaire and answer the above research questions, the reliability analysis, KMO test and Bartlett spherical test of the data were firstly carried out in this study, and the common method deviation test was also carried out. Secondly, the correlation analysis of each variable is carried out. In addition, the control variables and independent variables were put into the linear regression model successively, and the influence of independent variables on dependent variables was obtained. Finally, multiple linear regression was used to test the mediating effect on the basis of the control variables.

### 3.2. Measures

Dependent variable: political identity. This study investigates the current situation of political identity of China’s Small-town Youth from three dimensions: political interest identity, political system identity and political value identity. Among them, political interest identification investigates the satisfaction of China’s Small-town Youth on economic income and hometown construction and development. Political system identification investigates the understanding and support of China’s Small-town Youth to the leaders and staff of political organizations. Political value identification investigates the understanding and support of China’s Small-town Youth on the significance and value of the implementation of national policies, socialist core values, patriotism and other core values. A five-level Likert scale is used for measurement (The values are assigned sequentially from 1 to 5, with 1 indicating completely disagree and 5 indicating completely agree).

Independent variable: short video usage. It is mainly measured by asking respondents “the use of short videos in the past year,” including the overall frequency of short video browsing, liking, commenting, forwarding and collecting. A four-level Likert scale is used to assign values according to “almost every day,” “often,” “occasionally” and “hardly at all.”

Intermediary variables: (1) media trust. Based on the subjective evaluation of respondents on the credibility of media content, this study investigates the trust degree of China’s Small-town Youth in short videos. By asking “how credible do you think the information on the short video platform is,” a five-level Likert scale is used to measure the information (1 = downright distrust, 5 = absolutely trust). (2) Political trust. It mainly investigates the overall level of trust of China’s Small-town Youth in government agencies and public officials, including 11 options such as courts, procuratorates, public security organs, the Party Central Committee and the central government, provincial government, municipal government, county government, township government, neighborhood committee, village committee, and social organizations including trade unions, disabled persons’ federations, literary and cultural federations, women’s federations, etc. Measure with Likert five level scale (1 = downright distrust, 5 = absolutely trust).

Control variables: in addition to short video usage, media trust and political trust, some demographic variables also affect the political identity of China’s Small-town Youth. Referring to previous studies, the gender, age, education level, political outlook, occupation type, birthplace, and current living status of the respondents are included in the statistical analysis model as control variables.

## 4. Results

### 4.1. Reliability and validity test

SPSS25.0 was used to process and analyze the data, and the reliability and validity of the variables were tested: Cronbach’s Alpha value of all variables were greater than 0.7, and KMO measure value was 0.864 (*p* < 0.05). The reliability and validity were favorable and acceptable, so data quality was reliable. Further, principal component analysis was conducted on the questionnaire items, and the analysis results were as follows.

### 4.2. Reliability and validity test

Common method bias refers to the artificial covariation between predictive and outcome variables due to the same data source or rater, the same measurement environment, the project context, and the characteristics of the project itself. Controlling such systematic bias can effectively reduce the potential misleading of the conclusion. In the first step, eight factors with eigenvalues greater than 1 were identified using Harman single factor test. The variance explanation percentage of the first common factor was 29.62%. In general, when the variance explanation percentage of the first common factor was less than 40%, it could be considered that there was no serious common method bias. Secondly, partial correlation analysis is used to further test. The first common factor without rotation was isolated through exploratory factor analysis, and then the partial correlation between independent variable and dependent variable was investigated after controlling the common factor. If the original correlation coefficients are still significant after the analysis according to the above steps, the influence of the common method bias can be ignored. After controlling for the first common factor precipitated in this study, the correlation between short video use and political identity was still significant, indicating that there was no serious common methodology bias in this study.

### 4.3. Correlation analysis of short video use, media trust, political trust, and political identity

The results show that the average score of using frequency of short videos among China’s Small-town Youth is 2.96, and the standard deviation is 1.043, indicating that the respondents’ overall use of short videos is close to regular use. The average score of political identity of China’s Small-town Youth is 3.58, and the standard deviation is 0.543, indicating that the majority of respondents have a positive political attitude. Among them, political system identification (M = 3.74) is higher than political interest identification (M = 3.44) and political value identification (M = 3.56). The average score of media trust is 3.05 and the standard deviation is 0.708. The average score of political trust is 3.89, and the standard deviation is 0.702.

There is a significant correlation between the use of short videos of China’s Small-town Youth and three variables: political identity (*r* = 0.121, *p* = 0.006 < 0.01), media trust (*r* = 0.301, *p* = 0.000 < 0.01), and political trust (*r* = 0.122, *p* = 0.005 < 0.01). There is a significant correlation between media trust and political identity (*r* = 0.256, *p* = 0.000 < 0.01), and political trust (*r* = 0.165, *p* = 0.000 < 0.01). There is a significant correlation between political trust and political identity (*r* = 0.542, *p* = 0.000 < 0.01). All variables have correlation, which is suitable for further regression analysis and intermediary effect test.

### 4.4. Multiple regression analysis of short video use on political identity of China’s small-town youth

In order to further reveal how China’s Small-town Youth’s short video use affects their political identity, this study takes demographic variables as control variable, short video use frequency as predictor variable, and the dependent variables are political interest identity, political system identity, and political value identity, respectively. Forced entry method is used to conduct multiple regression analysis on the above variables, to investigate the effects of short video use on different levels of the three dimensions of political identity.

The regression results showed that the frequency of short video use had a weak promoting effect on political interest identification, and the regression coefficient β value was 0.077, but the regression results were not significant (*p* > 0.05). However, there were significant positive effects on political system identity and political value identity (*p* < 0.05). The regression coefficient β value of short video use and political system identity was 0.119, and that of political value identity was 0.094. The result shows that the use of short videos to improve the political identity of China’s Small-town Youth mainly depends on the political system and value identity dimensions. On the basis of the control variable module, the main effect module increases the variation of the three dependent variables by 0.006, 0.014 and 0.009, respectively.

In the part of control variables, the gender, living status and occupation type of China’s Small-town Youth have significant influence on their political identity, especially the influence of occupation type cannot be ignored. The three dimensions of political identity of Party and state organs and public institution workers are generally higher than those of other occupations. Compared with the three dimensions of political identity that are affected by occupation type, the gender and residence status of China’s Small-town Youth only affect political value identity and political interest identity, respectively. The political value identification of male China’s Small-town Youth is higher than that of female. China’s Small-town Youth who stay in big cities to work and now live in the first and second tier cities generally have higher political interests than those who return from big cities. In addition, the political status and education level of China’s Small-town Youth have no significant influence on their political identity.

### 4.5. The intermediary effect test of media trust and political trust

**FIGURE 1:**
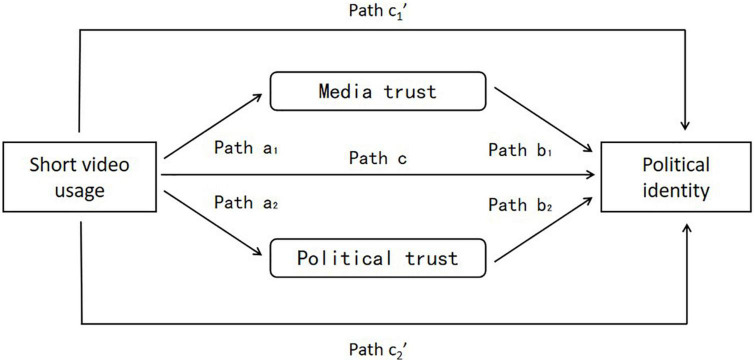
Model of intermediary effect test.

According to the above analysis, there is a significant correlation between the variables, which meets the premise of the mediating effect test. According to the traditional mediating effect test method, under the control of demographic variables, this study uses multiple regression to test the mediating effect of media trust and political trust on the relationship between short video use and political identity of China’s Small-town Youth in five steps, and the test model is shown in Figure 1 and Table 1.

**TABLE 1:** Variable measures.

Variables	Items	Factor loadings	Cronbach’s Alpha	Scale source
Short video use	A1. Frequency of viewing short videos	0.55	0.76	Ma and Wang, 2015; Yang and Sun, 2019
	A2. Frequency of liking short videos	0.78		
	A3. Frequency of commenting on short videos	0.79		
	A4. Frequency of retweeting short videos	0.74		
	A5. Frequency of collecting short videos	0.76		
Media trust	B1. I trust the information on short video platforms			Ma and Wang, 2015
Politics trust	C1. I trust the court and its public officials	0.86	0.92	Mei and Tao, 2018; Wang and Jin, 2019
	C2. I trust the public Prosecutor’s office and its public officials	0.83		
	C3. I trust the public security authorities and their public officials	0.42		
	C4. I trust the party and central governments and their public officials	0.74		
	C5. I trust the provincial government and its public officials	0.86		
	C6. I trust the municipal government and its public officials	0.90		
	C7. I trust county governments and their public officials	0.90		
	C8. I trust the township government and its public officials	0.90		
	C9. I trust the resident council and its public officials	0.82		
	C10. I trust the Village Council and its public officials	0.79		
	C11. I trust organizations such as trade unions, disabled persons’ associations, cultural associations, women’s associations and their public officials	0.83		
Political identity	D1. The development of the country is closely related to me	0.69	0.85	Kong, 2007; Fang et al., 2013
	D2. When personal interests and national interests conflict, personal interests must be unconditionally subordinated to national interests	0.68		
	D3. I agree with the Chinese political system, which has promoted China’s development	0.88		
	D4. I support China’s political system, which reflects the superiority of China’s socialist system	0.86		
	D5. I think the Chinese political system has legitimacy and authority	0.88		
	D6. I agree with the socialist core values emphasized by the Party and government	0.60		
	D7. I don’t think there are so-called “universal values” such as freedom, democracy and human rights in the world	0.64		
	D8. I think China must adhere to Marxism-Leninism and Mao Zedong Thought as its guide and follow the road to socialism	0.67		
	D9. I support that teachers should never be allowed to make statements critical of the party and government in the classroom	0.61		
	D10. I believe that groups and troublemakers who challenge the authority of the government and the existing social order must be severely punished	0.75		
	D11. I think I am capable of participating in political or public affairs	0.37		
	D12. I think government leaders are like the parents of an extended family, and anyone should obey them	0.64		
	D13. I believe that China’s diplomatic problems such as territorial and trade disputes are mainly caused by other countries provoking them in the first place	0.59		
	D14. I believe that Taiwan should be reunified by force if conditions permit	0.43		
	D15. I think if you are patriotic, you must boycott Japanese and American goods	0.76		
	D16. I believe that foreign hostile forces are determined to kill us and that they are behind many of China’s problems	0.63		
	D17. I think I must be a powerful leader when I have the opportunity to make others obey me	0.79		

It is found that the use of short videos by China’s Small-town Youth can positively predicts political identity (β = 0.111, *p* < 0.01), and H1 is supported. Meanwhile, the test data shows that the use of short videos is positively correlated with media trust and political trust, and the higher the frequency of short videos, the stronger the media trust and political trust of China’s Small-town Youth. Both media trust and political trust are positively correlated with political identity. When the mediating variable media trust was included in the model, the effect of short video use on political identity was significantly weakened, the regression coefficient decreased from 0.111 to 0.052, and the direct path changed from significant to insignificant. When the mediating variable political trust was included in the model, the regression coefficient decreased to 0.049, and the direct path also changed from significant to insignificant (Table 2). This indicates that short video use indirectly affects the political identity of China’s Small-town Youth through media trust and political trust, respectively. Both media trust and political trust play a complete mediating role between short video use and political identity, and H2 and H3 hold (shown in Tables 3, 4).

**TABLE 2:** Correlation of core variables.

		Short video usage	Media trust	Political trust	Political identity
Short video usage	Pearson correlation	1	0.301**	0.122**	0.121**
	Sig.		0.000	0.005	0.006
Media trust	Pearson correlation	0.301**	1	0.165**	0.256**
	Sig.	0.000		0.000	0.000
Political trust	Pearson correlation	0.122**	0.165**	1	0.542**
	Sig.	0.005	0.000		0.000
Political identity	Pearson correlation	0.121**	0.256**	0.542**	1
	Sig.	0.006	0.000	0.000	

**Indicates significance at the 0.01 level.

**TABLE 3:** Multiple regression analysis of short video use on political identity of China’s small-town youth (standard regression coefficient).

Predictive factor	Political interest identity				Political system identity				Political value identity			
	**M1**		**M2**		**M4**		**M5**		**M7**		**M8**	
	**β**	***p***	**β**	***p***	**β**	***p***	**β**	***p***	**β**	***p***	**β**	***p***
Dwelling state	-0.105**	0.015	-0.109*	0.012	–0.015	0.731	-0.020	0.640	-0.049	0.247	-0.053	0.207
Gender	-0.085	0.053	-0.084	0.056	–0.063	0.160	-0.060	0.173	-0.142**	0.001	-0.140**	0.001
Level of education	-0.059	0.222	-0.060	0.216	–0.019	0.696	-0.020	0.679	-0.041	0.393	-0.042	0.382
Politics status	-0.072	0.129	-0.068	0.154	–0.078	0.106	-0.071	0.138	-0.054	0.248	-0.049	0.296
Vocational type	-0.164***	0.000	-0.160***	0.000	–0.129	0.005	-0.122**	0.008	-0.210***	0.000	-0.204***	0.000
*R* ^2^	0.058				0.026				0.082			
Short video usage			0.077	0.071			0.119**	0.006			0.094*	0.025
△*R*^2^			0.006				0.014				0.009	
*R* ^2^			0.064				0.040				0.091	
Adjusted *R*^2^			0.053				0.029				0.081	
*F*	6.425***		5.922***		2.728*		3.573**		9.303***		8.652***	

***, **, *Indicate significance at 0.001, 0.01, and 0.05 levels, respectively.

**TABLE 4:** The intermediary effect test of media trust and political trust.

Inspection steps	Dependent variable	Independent variable	SE	β	t	*p*
1 (Path c)	Political identity	Short video usage	0.034	0.111	2.617**	0.009
2 (Path a_1_)	Media trust	Short video usage	0.044	0.290	7.090***	0.000
3 (Path b_1_) (Path c_1_′)	Political identity	Media trust	0.034	0.203	4.569***	0.000
		Short video usage	0.035	0.052	1.188	0.236
4 (Path a_2_)	Political trust	Short video usage	0.046	0.116	2.655**	0.008
5 (Path b_2_) (Path c_2_′)	Political identity	Political trust	0.027	0.533	15.021***	0.000
		Short video usage	0.029	0.049	1.372	0.171

***, ** and *Indicate significance at 0.001, 0.01, and 0.05 levels, respectively.

## 5. Conclusion and discussion

### 5.1. Conclusion

Based on the data results, this study presents the following four key findings:

First, the use of short videos is helpful to improve the political system identity and political value identity of China’s Small-town Youth. The use of short videos has a vitally positive impact on the political identity of China’s small-town youth, among which the positive impact on political system identity and political value identity is significant and the impact on political interest trust is weak. Among the three dimensions of political identity, interest identity is the starting point and foundation of political systems and value identity. However, the low impact of short videos on political interest trust has a realistic rationale. At present, there are differences in political identity among China’s small-town youth. Some young people’s hopes for future development and quality of life coexist with uncertainties, and they face survival difficulties in work and life, leading to a lack of identity. Although it is difficult to satisfy the material needs of reality only through the spiritual world of short videos, various images can be displayed on the short video platform to express emotions, express the yearning for a better life, or make informational compensation through the positive topics pushed by short videos (Li et al., 2022). This further indicates that short video ideological guidance should focus on the promotion of the superiority of the political system and the transmission of mainstream values, and the identification of political values is also helpful to promote the identification of the country, political party, policy system, and the observance of social and political order.

Second, China’s Small-town Youth promote political identity shaping mainly through the political use of short videos. The use of short videos significantly promotes the political identity of China’s small-town youth. However, not all content on short video platforms can effectively improve the political identity of China’s small-town youth. At present, government departments at all levels and mainstream media have entered the short video platform to seize the public opinion field of short video (Peng et al., 2019). Short video is not only a place for recreation and entertainment, but it is also a diverse and unintentional field of public opinion (Cui and Tong, 2022), which has become the field of many public events reporting and open discussion. From government micro-blogs to WeChat to short videos, short videos of government affairs are the product of the government’s active adaptation to the information supply and demand in the Internet era. Mobile short video has become an important field of public opinion in the new era (Cui and Tong, 2022; Peng et al., 2022). This research shows that the small town youth group is not numbly addicted to the pan-entertainment information flow but actively uses the short video platform, makes full use of its public communication function, and actively engages in political information contact. Hard news short video has sufficient market demand.

Third, media trust and political trust play an intermediate role in the influence of short video use of China’s Small-town Youth on political identity.

Third, media trust and political trust directly affect the formation of political identity among China’s small-town youth. The higher the media trust and political trust of small town youth, the higher their political interest trust, political system identity, and political value identity will be, and the better their political identity will be. This is consistent with previous research findings that media trust influences people’s views on socio-political democracy (Ognyanova and Ball-Rokeach, 2015). The higher the trust in the media, the more likely people are to believe the information the media conveys and the more confident they are about the country and government decisions. In particular, the media trust and political trust of short government affairs videos will significantly affect the communication effect of short government affairs videos and improve psychological cognitive recognition and trust of official media and political systems (Peng et al., 2022).

In the influence of short video use on political identity, media trust and political trust play an important mediating role. The higher the frequency of short videos, the higher the media trust and political trust of China’s small-town youth, and the more obvious the positive impact on political identity. Media trust affects the audience’s acceptance of short video content, while political trust directly affects the public’s political attitude. According to the theory of political socialization, both of them are influenced by the use of short videos, and together with the latter, they constitute an interactive system that affects political identity. The lower the political information contact cost, the more effectively people participate in public affairs discussion and political participation through short video, and the more inclined they are to change from passive political identity to active political identity (Hou et al., 2021). Therefore, in order to enhance the positive impact of short video use on the political identity of China’s small-town youth, it is necessary to continuously improve and enhance the media trust of small-town youth in short video platforms and their political trust in the country and government.

Fourth, dwelling state, gender and occupation affect the political identity of China’s Small-town Youth not the cultural identity. The occupation type of China’s small-town youth has influence on the three dimensions of political identity. Some small town youth have unclear career planning, worry about the surrounding environment and their own situation, and have low social security. Compared with stable occupations such as civil servant, small-town youth engaged in other occupations have serious anxiety about unemployment, which leads to a decrease in their subjective wellbeing and satisfaction with the country, society, and political party. Moreover, with the increase in occupational instability and the decrease in compensation, their political identity also decreases. This has to do with the basic national conditions in China, where people have a higher sense of gain within the system. The gender and dwelling state of China’s small-town youth, respectively, affect their political value identity and political interest trust. Male Chinese small-town youth have a stronger political identity than females. The political interest trust of “ranger type” (China’s small-town youth who stay in big cities and struggle and now live in first and second-tier cities) is generally higher than that of “reflux type” (China’s small-town youth who return home from big cities).

### 5.2. Discussion on ideology, identity, and gender

On the whole, the research results refute the pessimistic view of the ideological threat of short videos; that is, the inference that the thoughts of China’s small-town youth will deviate from the right track due to exposure to inferior content has not been confirmed, and the overall use of short videos by China’s small-town youth promotes the shaping and promotion of political identity (Chen, 2021). Although short videos appear to be the result of pan-entertainment in a fast-paced era (Xiao et al., 2019), they abandon complex narrative modes and editing techniques and deepen the public’s understanding and cognition of political systems and values through emotional communication (Cao, 2021; Cui and Tong, 2022). Short video has a strong impact on vision and hearing and has more rendering power than other media. Short video has distinct advantages as a tool for guiding ideas, particularly for guiding the values of society’s middle and lower classes.

At present, it is common for China’s small-town youth to “retire to the countryside” (Guo, 2020), which is highly consistent with the overall vision of China’s rural revitalization strategy. However, in the process of participating in the construction of the countryside, complicated “role expectations,” “shallow” social integration and a fragile social support system can easily lead to deviations in the ideals of small town youth. The overall quality of life and material conditions of big cities are better than those of small town;, the social security system is more perfect;, and the young people in small towns will be more willing to identify with the ruling party (Liu, 2012). At the individual level, people’s identification of political interests is more determined by their own experience and observation of the surrounding environment. Because of gender discrimination in society, female youth in small towns have little political identity. Walzer (1990) believed that political identity and cultural identity are not necessarily mutually exclusive, and cultural identity can help people with a sense of ethnic identity find their spiritual destination. Cultural identity should be prevented from evolving into narrow tribalism. Political identity is the basic condition for the existence of any country, but there is no need to threaten the survival of disadvantaged cultural groups with positive assimilation policies (Walzer et al., 1982). Therefore, cultural identity and political identity should coexist, and they should interact and grow. Although the Chinese government has been emphasizing and educating young people to identify with Chinese culture, it can be seen from in-depth interviews that cultural identity cannot effectively promote the political trust of small town youth, but plays an obvious role in national identity.

### 5.3. Theoretical contribution and practical implication

In recent years, studies of different orientations have drawn different conclusions on the direction of the effect of Internet media on political identity (Cao, 2021; Chen, 2021; Cui and Tong, 2022). Although the positive effect of the use of traditional media or official media on political attitudes is widely recognized, there are still discussions on the negative, ineffective, and positive positions of the media influence of the Internet or social media. At the theoretical level, this study reveals the inner psychological mechanism of the formation of political identity in China’s small-town youth and explores how the use of short videos affects their political identity. Its theoretical contribution is to find the promotion effect of short video use on the political identity of China’s small-town youth and clarify the mechanism of short video use on political identity from the perspective of media trust and political trust. On this basis, this study puts forward the following thoughts on how to improve the political identity of small-town youth through the short video platform:

First and foremost, consideration should be given to the far-reaching importance of a short video platform in shaping the political identity of small-town youth. For one thing, we should standardize the media production content of short video platforms, strictly formulate and implement the user management treaty of each platform, call on short video platforms to jointly advocate positive value orientation, and build a legal and compliant short video public opinion field in the new era. For another, the platform algorithm mechanism should be used rationally to encourage the push support of high-quality short video content while avoiding the dissemination of undesirable content that can exacerbate social conflicts and instill negative values (Xiao et al., 2019; Li et al., 2022).

Secondly, the short video platform for government affairs should pay attention to the political participation significance of short video interaction and improve its political communication efficiency (Hou et al., 2021). The government and mainstream media should pay attention to the positive impact of short videos on small-town youth political identity. On the one hand, based on the perspective of government services, through emotional commitment and interaction, we should improve the stickiness of short videos of small town youth for political purposes. On the other hand, we should optimize the content of political information, emphasize the professionalism, authority, and appeal of the content, and form a benign public discussion atmosphere in the field of public opinion about short videos.

Thirdly, high media trust and political trust are the basis for the cultivation and promotion of political identity (Hou et al., 2021), which should be transformed from management logic to governance logic and strengthen the coordination and interaction among the government, platform, and audience. The supervision department of the short video platform should strengthen the examination of the authenticity and orientation of the content to enhance trust in the short video platform. Government departments should establish a good image in the administrative process, using the short video platform of government affairs to strengthen government agenda setting and win the trust and recognition of small town youth.

Ultimately, we should pay attention to the value guidance of the small-town youth who feel at a loss in China’s urban-rural dualization. Relevant support measures for small town youth groups are implemented to enhance their political identity (Chen, 2021). At the same time, the short video platform is used to guide small town youth to correct their cognition of objective reality, actively adjust and escape negative mentality, and enhance their sense of responsibility and mission to promote rural revitalization and urban-rural integration development.

### 5.4. Limitations and future research

There are still limitations in this study. First, this study mainly investigates the impact of short video usage frequency on the political psychology of China’s Small-town Youth. The relationship between short video use appeal, content preference, participation and political psychology is worth further exploring. Second, in the Polymedia Environment, the media contact channels of China’s Small-town Youth are diversified, and the impact of different types of media use on their political psychology and their interaction effects need to be further studied. Third, this study only focuses on China’s Small-town Youth. In the future, a comparative study on the impact of media use of Small-town Youth on their political attitudes in different countries can be carried out.

## Data availability statement

The original contributions presented in this study are included in the article/supplementary material, further inquiries can be directed to the corresponding authors.

## Author contributions

JQ and QD contributed to the research design and implementation. QD and XS collected the data. YD and BZ analyzed the results. All authors contributed to the writing of the manuscript.
